# Deficiency of the myogenic factor MyoD causes a perinatally lethal fetal akinesia

**DOI:** 10.1136/jmedgenet-2015-103620

**Published:** 2016-01-05

**Authors:** Christopher M Watson, Laura A Crinnion, Helen Murphy, Melanie Newbould, Sally M Harrison, Carolina Lascelles, Agne Antanaviciute, Ian M Carr, Eamonn Sheridan, David T Bonthron, Audrey Smith

**Affiliations:** 1 Yorkshire Regional Genetics Service, St. James's University Hospital, Leeds, UK; 2 School of Medicine, University of Leeds, St. James's University Hospital, Leeds, UK; 3 Genomic Medicine, Manchester Academic Health Science Centre, The University of Manchester, St Mary's Hospital, Manchester, UK; 4 Department of Paediatric Histopathology, Central Manchester University Hospitals NHS Foundation Trust, Manchester, UK

**Keywords:** <i>MYOD1</i>, perinatal lethal, fetal akinesia, lung hypoplasia, exome sequencing

## Abstract

**Background:**

Lethal fetal akinesia deformation sequence (FADS) describes a clinically and genetically heterogeneous phenotype that includes fetal akinesia, intrauterine growth retardation, arthrogryposis and developmental anomalies. Affected babies die as a result of pulmonary hypoplasia. We aimed to identify the underlying genetic cause of this disorder in a family in which there were three affected individuals from two sibships.

**Methods:**

Autosomal-recessive inheritance was suggested by a family history of consanguinity and by recurrence of the phenotype between the two sibships. We performed exome sequencing of the affected individuals and their unaffected mother, followed by autozygosity mapping and variant filtering to identify the causative gene.

**Results:**

Five autozygous regions were identified, spanning 31.7 Mb of genomic sequence and including 211 genes. Using standard variant filtering criteria, we excluded all variants as being the likely pathogenic cause, apart from a single novel nonsense mutation, c.188C>A p.(Ser63*) (NM_002478.4), in *MYOD1*. This gene encodes an extensively studied transcription factor involved in muscle development, which has nonetheless not hitherto been associated with a hereditary human disease phenotype.

**Conclusions:**

We provide the first description of a human phenotype that appears to result from *MYOD1* mutation. The presentation with FADS is consistent with a large body of data demonstrating that in the mouse, MyoD is a major controller of precursor cell commitment to the myogenic differentiation programme.

## Introduction

Lethal fetal akinesia deformation sequence (FADS; OMIM 208150) comprises a spectrum of clinically and genetically heterogeneous disorders that is most commonly detected prenatally, through the routine application of middle-trimester ultrasound scanning. Its clinical features, which are secondary to the lack of fetal movement, include intrauterine growth retardation, arthrogryposis and developmental anomalies such as lung hypoplasia, characteristic facies, cleft palate and cryptorchidism.^1^ Affected babies either die in utero or shortly after birth. The akinesia phenotype can be due to a genetic defect intrinsic to the fetus, but also to non-genetic causes such as maternal myasthenia gravis.

To date, >20 genes have been associated with fetal akinesia, representing all modes of inheritance. Many of these genes encode components of neuromuscular pathways, as described in the comprehensive review by Ravenscroft *et al*.^2^

The prenatal presentation of FADS complicates diagnosis. Although anatomical examination, especially using advanced ultrasonographic techniques,^3^ ^4^ can provide important diagnostic information regarding associated malformations such as facial clefting, prognosis cannot necessarily be inferred with certainty. Postmortem examination may result in a more precise pathological classification, but given the aetiological heterogeneity of FADS, a genetic diagnosis (preferably prenatally) remains highly desirable.

High-throughput DNA sequencing has broadened the scope of traditional hypothesis-free genetic investigations from the detection of structural variants using arrayCGH (or low-coverage whole-genome sequencing, ‘CNV-seq’) to encompass point mutation and small insertion/deletion variant detection using exome sequencing. The methods employed in large multicentre studies of human paediatric phenotypes (such as ‘Deciphering Developmental Disorders’^5^) are now also being applied to large-scale prenatal studies such as the Prenatal Assessment of Genomes and Exomes project.^6^ To perform new gene discovery, these projects rely on ascertaining a large number of trio samples (typically singleton affected cases and their parents) with similar phenotypes. The expectation is that more than one family within the cohort will have a disease-causing mutation in the same gene, allowing a novel gene function to be attributed. Despite the large size of these data sets, it is frequently the case that similarly affected patients can only be identified outside of the study cohort.

The integration of more ‘traditional’ types of genetic mapping data into the interpretation of genome-wide sequencing data sets permits an alternative approach, in which candidate genes can be filtered using information from a single family. This permits small-scale gene discovery programmes, which may arise directly from data sets accrued in diagnostic laboratories, without the need for large and expensive recruitment programmes.^7^ Filtering on the basis of identity by descent, either explicit from family history, or implicit based on the rarity of a disease or isolation of an ethnic group, is one very powerful genetic approach.

Here, we report a consanguineous Caucasian family in whom three affected individuals, from two sibships that share the same mother, presented with features consistent with FADS. Exome analysis with homozygosity-based filtering resulted in the identification of *MYOD1* as a new gene underlying FADS.

## Methods

Consultant clinical geneticists clinically examined and reviewed postmortem examination reports that had been undertaken on the deceased babies. DNA was isolated using phenol/chloroform extraction or standard salting out (for blood) protocols, following written informed consent.

Illumina-compatible exome sequencing libraries were generated for samples II:2, III:1 and III:4 using SureSelect v.5 reagents, following manufacturer's protocols throughout (Agilent Technologies, Wokingham, UK). A low DNA input protocol was followed to create the sequencing library for individual III:2 using alternative library preparation reagents (New England Biolabs, Ipswich, UK). The four exome-enriched libraries were pooled in equimolar concentration with either 3 (III:2) or 4 (II:2, III:1 and III:4) other sequencing libraries prepared as part of the laboratory's standard diagnostic testing workflow. Sequencing of each pool was performed using both lanes of a HiSeq2500 rapid mode flow cell, generating paired-end 100 bp sequence reads (Illumina, San Diego, California, USA).

Despite the poor quality material obtained for DNA extraction from the affected individuals (and low DNA yield for sample III:2), the per-patient sequencing summary metrics were comparable to those obtained from the exome library of the unaffected mother (whose DNA source was peripheral blood lymphocytes, our preferred specimen). Notably a minimal number of reads were adaptor trimmed (∼3.5%) or identified as duplicate read pairs (∼12.4%) (see online supplementary table S1).

Data processing was performed using an in-house informatics pipeline. Briefly, raw sequence data were converted from bcl to FASTQ.gz format and demultiplexed using CASAVA V.1.8.2. Adaptor sequences were trimmed from per-patient sequence reads using Cutadapt V.1.7.1 (https://code.google.com/p/cutadapt/)^8^ before being aligned to the human reference genome (hg19) using bwa V.0.7.12 (http://bio-bwa.sourceforge.net).^9^ Sam to bam conversion, duplicate read removal and read sorting based on mapped read coordinates were performed using Picard V.1.129 (http://picard.sourceforge.net). The Genome Analysis Toolkit (GATK) V.2.3-4Lite was used to perform indel realignment, base quality score recalibration and variant discovery, which resulted in variants being saved in variant call format (VCF)^10^ before being annotated with positional, functional and frequency data using Alamut Batch standalone V.1.4.0 (Interactive Biosoftware, Rouen, France). Programs ancillary to the automated pipeline were used to interrogate these data, namely Agile MultiIdeogram (http://dna.leeds.ac.uk/agile/AgileMultiIdeogram), which was used to determine regions of autozygosity from the per-patient VCF files, and Agile Exome Filter (http://dna.leeds.ac.uk/agile/AgileExomeFilter), which was used to filter Alamut Batch annotated variant reports using positional and functional parameters.^7^ Interrogation of the Exome Aggregation Consortium data set was performed using the ExAC Browser (http://exac.broadinstitute.org). Manual inspection of aligned sequence reads was performed using the Integrative Genome Viewer V.2.3.57.^11^ The number of sequence reads mapping to each target base was calculated using the GATK DepthOfCoverage walker.

To confirm and genotype the putative pathogenic *MYOD1* variant, a 383 bp amplicon was designed and optimised before being Sanger sequenced using an ABI3730 following manufacturer's protocols (Applied Biosystems, Paisley, UK). Each reaction consisted of 0.5 μL of genomic DNA (∼500 ng/μL), 11 μL of Megamix (Microzone, Haywards Heath, UK), 1 μL of 10 pmol/μL forward primer (dTGTAAAACGACGGCCAGTACGACTTCTATGACGACCCG) and 1 μL of 10 pmol/μL reverse primer (dCAGGAAACAGCTATGACCCTGGTTTGGATTGCTCGACG). Both primers contained universal sequencing tags (underlined) to allow Sanger sequencing according to our standard laboratory workflows. Thermocycling conditions consisted of 5 min at 94°C, followed by 30 cycles of 94°C for 30 s, 55°C for 60 s and 72°C for 45 s, before a final extension step at 72°C for 5 min. Sequence chromatograms were viewed using Mutation Surveyor V.3.2 (SoftGenetics LLC, State College, USA).

## Results

**Figure 1:**
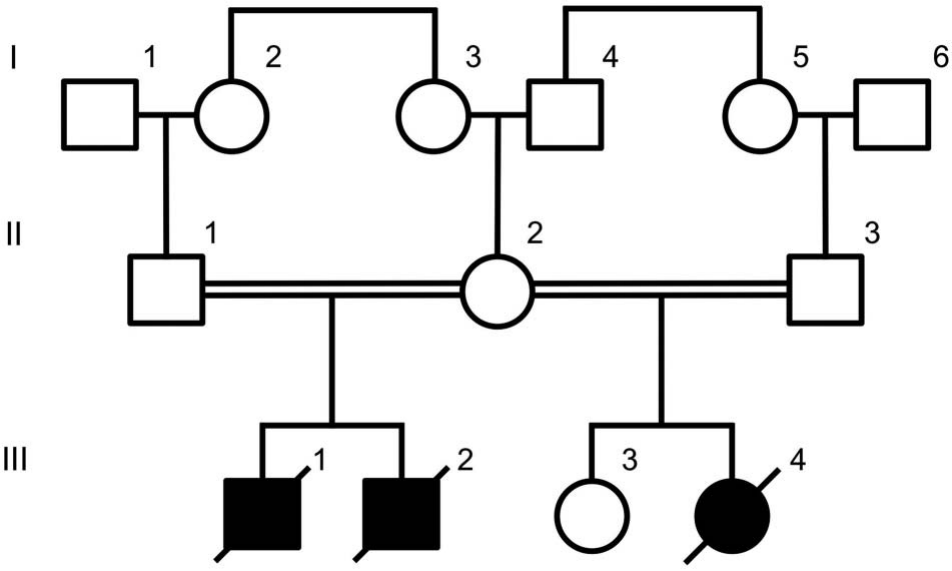
A pedigree showing the relationship between affected (shaded symbols) and unaffected (outlined symbols) individuals.

The pedigree structure of the family is shown in figure 1. Two affected male babies were born to a first-cousin consanguineous Caucasian couple.

The first child (III:1) was delivered following spontaneous labour at 35^+5^ weeks. During the pregnancy, a cystic hygroma had been noted at 12 weeks, which subsequently resolved, and polyhydramnios developed during the third trimester. The baby's birth weight was 1.87 kg (2nd to 9th centile) and his occipitofrontal circumference (OFC) was 33.0 cm (50th centile). Apgar scores were 1 at 1 min and 1 at 5 min. He required extensive resuscitation and was ventilator dependent. He had numerous episodes of oxygen desaturation and on day 2 failed to respond to an acute resuscitation for a further hypoxic episode and died. A postmortem examination noted dysmorphic features that included downslanting palpebral fissures, a small chin and square forehead. The toes and fingers were overlapping and the fists were held in a clenched position. The testes could not be palpated in the scrotum. There was a midline posterior cleft palate and lung hypoplasia. (The right and left lung weights were 15.1 and 11.5 g compared with a normal combined weight of 34.5 g.) There was a right-sided diaphragmatic eventration, although no hernia was identified. There was a mild degree of bilateral renal pelvis distention, more severe on the left (1.3 cm in maximum dimension). Microscopically, the fundamental structure of the lungs was normal, but there was a degree of pulmonary hypoplasia. The costochondral junction appeared irregular, and there was centrilobular congestion of the liver.

**Figure 2:**
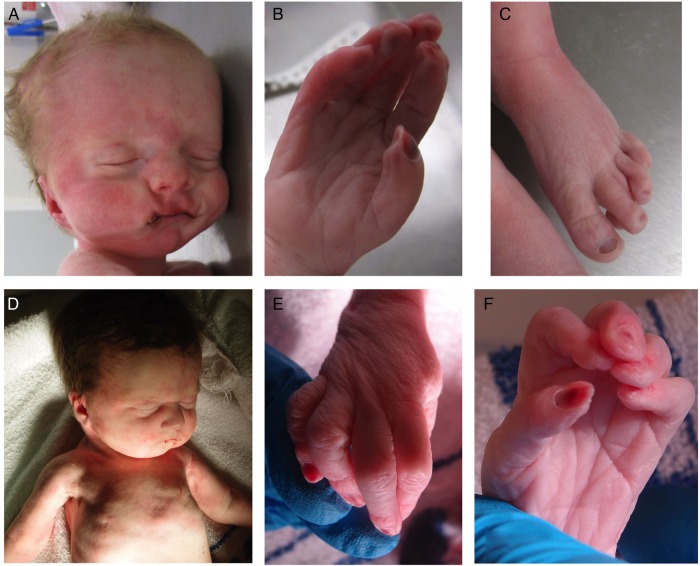
Affected infant III:2 showing (A) facial dysmorphism including a tall forehead with bitemporal narrowing, a long philtrum and a small chin; (B) the right hand showing long tapered fingers with contractures and (C) the left foot with overlapping toes. Affected infant III:4 showing (D) facial dysmorphism including a square forehead and small chin; apparent deficiency of pectoralis and proximal limb musculature (E and F) contractures of the proximal interphalangeal joints in the right and left hand, respectively.

The second child (III:2) was born at 35^+1^ weeks following spontaneous labour. During the pregnancy, serial growth scans had detected a right duplex kidney and increased liquor at 28 weeks. A scan at 34^+2^ weeks revealed polyhydramnios and gross right-sided hydronephrosis. The baby's birth weight was 1.80 kg (2nd centile) and his OFC was 33.5 cm (75th centile). Apgar scores were 1 at 1 min and 1 at 5 min. The baby died shortly thereafter, following unsuccessful resuscitation attempts. A postmortem examination noted dysmorphic features that included a tall forehead with bitemporal narrowing, a long philtrum and a small chin (figure 2A). He had long tapered fingers with contractures and overlapping toes (figure 2B, C). A single testis was palpable. There was a midline posterior cleft palate, and the right and left lungs were both extremely small (7 and 5.75 g). There was no obvious diaphragmatic hernia, but the domes were very high with extremely small pleural cavities. The right renal pelvis was extremely dilated with an obvious pelviureteric junction obstruction; the left kidney was normal.

The mother next delivered a third baby (III:3) whose father (II:3), a first-cousin relative, was different to that of her previously described affected offspring. The child was healthy.

A further daughter, III:4, was delivered at 37 weeks gestation, following a spontaneous labour. A cystic hygroma had been identified at 12 weeks. Chorionic villus sampling and prenatal arrayCGH analysis revealed no pathogenic CNVs. The baby's birth weight was 1.96 kg (0.4th to 2nd centile) and her OFC was 32.5 cm (25th–50th centile). Apgar scores were 1 at 1 min and 1 at 5 min. Despite intensive resuscitation, the baby died 30 min after delivery. Postmortem examination revealed a square forehead and small chin (figure 2D). There were contractures of the proximal interphalangeal joints from the 2nd to 5th fingers bilaterally (figure 2E, F). There was also bilateral knee flexion. There was a midline posterior cleft palate and extreme pulmonary hypoplasia. (Right and left lung weights were 8.2 and 6.5 g) There was no diaphragmatic hernia, but the domes were extremely high with small pleural cavities. The right and left kidney weights were 3.1 and 8.75 g (normal combined weight=17.4 g), but no obstruction was identified.

Postnatal arrayCGH analysis of samples III:1, III:2 and III:4 did not reveal any pathogenic variants that could account for the presenting phenotype. The sibling recurrence and reported consanguinity between parents suggested that the disorder was likely to be inherited in an autosomal-recessive manner.

DNA samples from all three affected individuals and their unaffected mother were subjected to exome sequencing.

**Figure 3:**
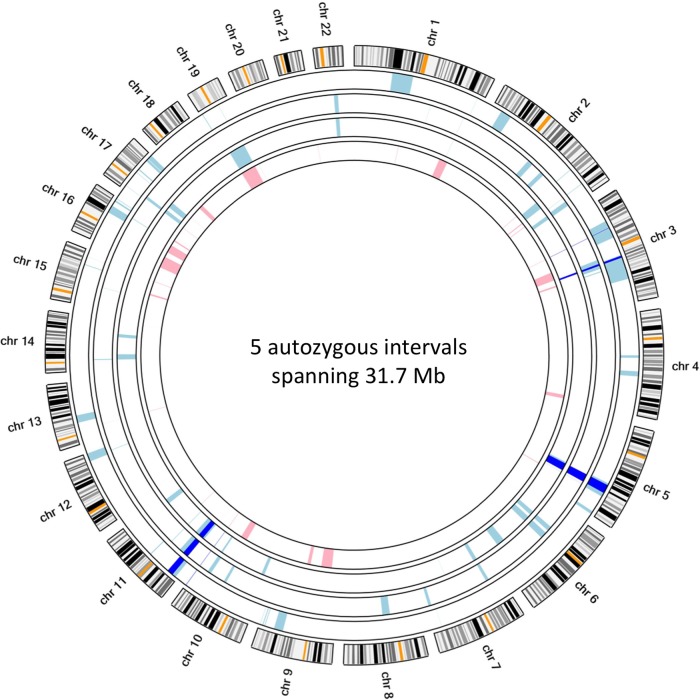
Autozygous intervals shared between all three affected individuals (dark blue) are shown with respect to per-sample autozygous intervals identified in affected individuals (light blue) and the unaffected mother (pink). The concentric circles, beginning from the interior, represent samples II:2, III:2, III:1 and III:4.

Having verified the quality control metrics of our data set (see ‘Methods’), we undertook autozygosity mapping using exome-wide SNP genotypes. Five genomic regions were identified to be identical by descent, which encompassed 31.7 Mb of genomic DNA sequence and 211 genes (figure 3 and online supplementary table S2). We analysed only homozygous variants located in these autozygous regions, thus reducing the mean total variant count from 35 820 to 269 variants per affected individual (table 1). Excluding common variants (those with a minor allele frequency >1% in either dbSNP or the ESP5400 data set) further reduced the mean number of variants to 12. To aid manual interpretation of the remaining variants, we retained only those variants located in coding regions or invariant splice sites. Of these, four variants were identified in a homozygous state in all affected siblings. Two of these were homozygous in the unaffected mother and on this basis were deemed to be non-pathogenic. A further two variants were heterozygous in the mother and warranted further investigation (see online supplementary table S3). The first of these was the missense variant *OTOG* c.3265A>G p.(Ile1089Val) (NM_001277269.1). Standard in silico tools suggested that this variant was unlikely to be pathogenic. (PolyPhen-2 result: benign; SIFT result: tolerated; AlignGVGD result: class C0.) Furthermore, *OTOG* has been previously described to cause autosomal-recessive deafness (OMIM: 604487) and did not offer an explanation for the clinical features of our patients. The second variant, *MYOD1* c.188C>A p.(Ser63*) (NM_002478.4), was predicted to be pathogenic and would likely lead to nonsense-mediated decay of the mRNA transcript. A thorough search of online databases did not identify this variant in any public databases. Further analysis of 61 486 exomes from the ExAC consortium revealed that homozygous loss-of-function (LOF) mutations had not been previously observed in *MYOD1*. In addition, *MYOD1* has not been previously classified as an LOF gene in data sets generated from previous LOF screens.^12–14^

**Table 1:** Filtering parameters per patient

Filtering criterion	III:1	III:2	III:3	Mean
Total variant count	36 290	36 304	34 867	35 820
Retain homozygous variants in autozygous intervals	258	269	280	269
Retain variants with a dbSNP/ESP5400 minor allele frequency ≤1%	14	13	9	12
Retain coding and invariant splice-site variants	7	7	5	6

The zygosity status and segregation of the c.188C>A *MYOD1* variant was confirmed by Sanger sequencing of six individuals. This included the second father II:3 and his unaffected daughter, III:3, both of whom were heterozygous carriers of the mutation (see online supplementary figure S1).

## Discussion

Here, we define by exome analysis of individuals affected with a perinatally lethal fetal akinesia syndrome, the first human phenotype that appears to be attributable to LOF of the myogenic factor MyoD. MyoD was the earliest described example of a developmental fate-controlling transcription factor^15^ and has been the subject of intensive study over a period of nearly 30 years. Remarkably though, LOF mutations in human *MYOD1* have not previously been identified.

The MyoD family of transcriptional regulators include two primary muscle lineage-determining factors *MyoD* and *Myf-5. MyoD* expressed from a constitutive promoter is able to convert a variety of different cell types into muscle-lineage cells in vitro.^16^ MyoD is a basic-helix-loop-helix domain-containing protein that functions by binding E-box sequences present in DNA.^17^ Histone acetyltransferases are then recruited to allow chromatin remodelling towards an environment that is conducive to active transcription.

Much work has been undertaken to elucidate the spatial and temporal differences in development between the MyoD/Myf-5 muscle regulatory factors. *MyoD* mutant mouse embryos show a 2-day delay in the development of hypaxial (limb and abdominal wall) muscle, whereas *Myf-5* mutants exhibit a similar delay in epaxial (paraspinal and intercostal) muscle development.^18^ However, lineage-tracing experiments combined with selective ablation of MyoD-expressing cells, using diphtheria toxin, indicate that the majority of myogenic progenitors pass through a MyoD+ cell fate and that myogenesis cannot be rescued by embryonic progenitor cells not expressing MyoD.^19^

It is, therefore, somewhat surprising that homozygous *MyoD* knockout mice are viable and fertile, produce seemingly normal amounts of the downstream myogenin protein and have no histologically detectable muscle abnormalities.^20^ Only when crossed with *Myf-5* knockout mice to create homozygous double mutants is a lethal phenotype (with complete absence of skeletal muscle) obtained; this suggests there is an element of functional redundancy between these two muscle regulatory factors.^21^ In contrast to these findings in the mouse, the family we report here suggests that lack of *MYOD1* is not compatible with postnatal life in humans.

Why human *MYOD1* mutations have not previously been recognised is uncertain, although recessive null alleles, as mentioned above, are clearly very rare. Prenatally and perinatally lethal disorders can be difficult to study because of limited availability and poor quality of DNA from affected individuals. This has led some to focus on analysis of parental samples in order to deduce pathogenic compound heterozygous genotypes in affected fetuses.^22^ ^23^

In the present case, we were able to perform direct analysis of affected individuals’ exomes, with interpretation aided by autozygosity mapping, as previously described.^24^ The autozygosity mapping and variant filtering we performed reduced the burden of variant interpretation to just two candidate disease-causing variants. In silico analysis of one of these variants (*OTOG* c.3265A>G NM_001277269.1) did not suggest a likely pathogenic effect. Furthermore, *OTOG* had previously been described to cause autosomal-recessive deafness.^25^ The second variant, *MYOD1* c.188C>A (p.Ser63*; NM_002478.4), is predicted to be pathogenic. In addition to introducing a stop codon within the N-terminal basic domain of the predicted protein, the location of this makes it highly likely that the mRNA transcript will be subject to nonsense-mediated decay. No previous homozygous LOF mutations have been identified in *MYOD1* in either of two large international projects. As this investigation is based on a single family, additional independent reports are required to confirm the proposed link between *MYOD1* mutations and FADS.

Our autozygosity analysis was performed by direct analysis of sequence variants extracted from whole-exome sequence. This is an approach that we have described previously;^24^ although it offers poorer resolution than whole-genome SNP array mapping, it frequently (as here) suffices and can be performed using data sets generated for routine diagnostic purposes. Of relevance to the present case, this method also avoids problems related to poor performance of array-based reagents in the face of DNA samples of poor quality or limited quantity.

## Supplementary Material

Web figure

Web table
