# Supplementary material for: Deficiency of the myogenic factor MyoD causes a perinatally lethal fetal akinesia
Source: J Med Genet. 2016 Jan 5;53(4):264–9. doi: 10.1136/jmedgenet-2015-103620 (PMC4819622; doi:10.1136/jmedgenet-2015-103620)

Supplementary Figure 1: Sanger sequencing confirmation of the nextgeneration sequencing identified variant c.188C>A (NM_002478.4) p.(Ser63*). The variant was confirmed to be homozygous in all affected siblings and heterozygous in the unaffected mother and unaffected father II:3.

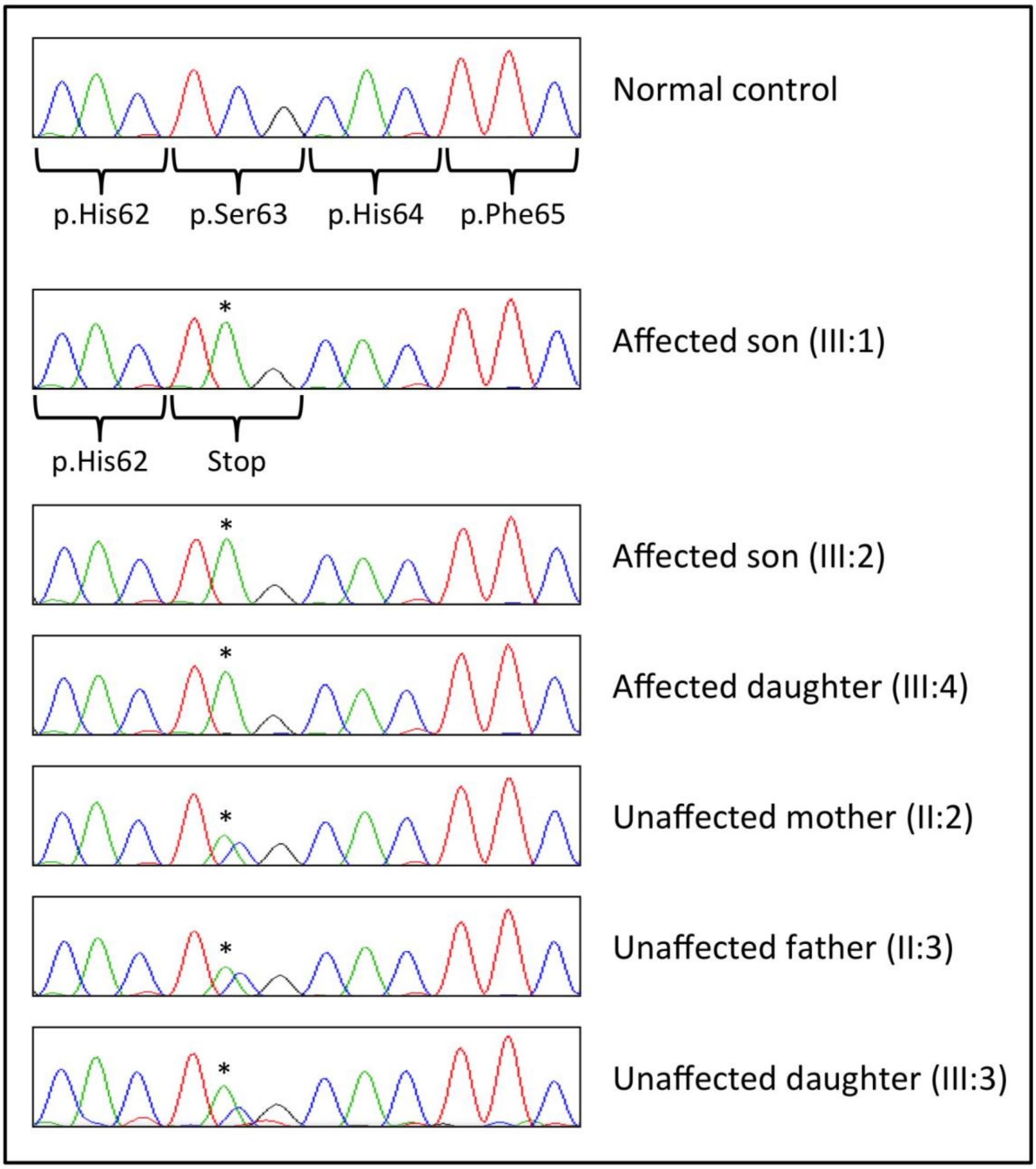

Supplement: Web figure [file jmedgenet-2015-103620-s1.pdf]
