# Supplementary material for: Deficiency of the myogenic factor MyoD causes a perinatally lethal fetal akinesia
Source: J Med Genet. 2016 Jan 5;53(4):264–9. doi: 10.1136/jmedgenet-2015-103620 (PMC4819622; doi:10.1136/jmedgenet-2015-103620)
Supplement: Web table [file jmedgenet-2015-103620-s2.pdf]

Supplementary Table 1: Sequencing summary metrics

Sample	Total raw reads	Adaptor trimmed reads (%)	Duplicate rate (%)	Mean insert size (bp)	Total mapped reads*	Exome mapped reads (%)	Percentage of target bases[§] sequenced	
							≥15×	≥30×
II:2	64,172,890	3.1	9.8	208	56,483,391	61.0	96.0	86.0
III:1	87,458,244	3.2	11.1	199	75,991,386	62.1	97.8	93.4
III:2	177,975,174	4.1	11.9	184	157,037,281	62.8	98.3	96.6
III:4	77,545,908	3.2	14.1	200	65,047,512	61.6	96.7	89.0

*Following duplicate removal. [§]Coding or invariant splice site nucleotides located in the autozygous region.

Supplementary Table 2: MultiIdeogram-identified autozygous regions

Chromosome	Start	Stop	Length (bp)	Genes* (n)
3	52,430,698	52,821,992	391,294	15
3	109,178,758	112,727,184	3,548,426	22
5	118,860,938	134,870,913	16,009,975	86
10	135,082,346	135,491,083	408,737	16
11	11,373,508	22,696,451	11,322,943	72
**Total**			**31,681,375**	**211**

*UCSC coding genes. Coordinates reported for hg19.

Supplementary Table 3: Homozygous filtered variants shared between three affected siblings and the zygosity status of the corresponding variants in their unaffected mother

RS number	Chr	Position	Gene	Transcript	c.Nomen, p.(Nomen)	Unaffected mother II:2		
						Zygosity	Non-reference allele read depth	Total read depth
RS40470	5	132,150,948	*SOWAHA*	NM_175873.4	c.1635T>G, p.(Phe545Leu)	Hom	0	93
RS56359117	11	17,615,244	*OTOG*	NM_001277269.1	c.3265A>G, p.(Ile1089Val)	Het	4	9
RS147517396	11	17,741,517	*MYOD1*	NM_002478.4	c.188C>A, p.(Ser63*)	Het	98	197
RS1136747	11	18,290,874	*SAA1*	NM_001178006.1	c.224T>C, p.(Val75Ala)	Hom	25¶	34

Chr: Chromosome. Hom: Homozygous. Het: Heterozygous. ¶: Manual inspection these data indicated the non-reference nucleotide containing reads were poor quality alignments whose sequence was derived from the *SAA1* pseudogene. The locus was therefore homozygous for the reference (T) nucleotide.
